# Development of a universal short patient satisfaction questionnaire on the basis of SERVQUAL: Psychometric analyses with data of diabetes and stroke patients from six different European countries

**DOI:** 10.1371/journal.pone.0197924

**Published:** 2019-10-17

**Authors:** Uwe Konerding, Tom Bowen, Sylvia G. Elkhuizen, Raquel Faubel, Paul Forte, Eleftheria Karampli, Tomi Malmström, Elpida Pavi, Paulus Torkki

**Affiliations:** 1 Department of Psychology and Psychotherapy, Witten/Herdecke University, Witten, Germany; 2 Trimberg Research Academy, University of Bamberg, Bamberg, Germany; 3 The Balance of Care Group, London, England, United Kingdom; 4 Institute of Health Policy & Management, Erasmus University Rotterdam, Rotterdam, The Netherlands; 5 Department of Physiotherapy, University of Valencia, Valencia, Spain; 6 Joint Research Unit in Biomedical Engineering (IIS La Fe- Universitat Politècnica de València), Valencia, Spain; 7 Department of Public Health Policy, School of Public Health, University of West Attica, Athens, Greece; 8 Department of Industrial Engineering and Management, Aalto University, Espoo, Finland; 9 Department of Public Health, Helsinki University, Helsinki, Finland; Hong Kong Polytechnic University, HONG KONG

## Abstract

**Objective:**

A short questionnaire which can be applied for assessing patient satisfaction in different contexts and different countries is to be developed.

**Methods:**

Six items addressing tangibles, reliability, responsiveness, assurance, empathy, and communication were analysed. The first five items stem from SERVQUAL (SERVice QUALity), the last stems from the discussion about SERVQUAL. The analyses were performed with data from 12 surveys conducted in six different countries (England, Finland, Germany, Greece, the Netherlands, Spain) covering two different conditions (type 2 diabetes, stroke). Sample sizes for included participants are 247 in England, 160 in Finland, 231 in Germany, 152 in Greece, 316 in the Netherlands and 96 in Spain for the diabetes surveys; and 101 in England, 139 in Finland, 107 in Germany, 58 in Greece, 185 in the Netherlands, and 92 in Spain for the stroke surveys. The items were tested by (1) bivariate correlations between the items and an item addressing ‘general satisfaction’, (2) multivariate regression analyses with ‘general satisfaction’ as criterion and the items as predictors, and (3) bivariate correlations between sum scores and ‘general satisfaction’.

**Results:**

The correlations with ‘general satisfaction’ are 0.48 for tangibles, 0.56 for reliability, 0.58 for responsiveness, 0.47 for assurance, 0.53 for empathy, and 0.56 for communication. In the multivariate regression analysis, the regression coefficient for assurance is significantly negative while all other regression coefficients are significantly positive. In a multivariate regression analysis without the item ‘assurance’ all regression coefficients are positive. The correlation between the sum score and ‘general satisfaction’ is 0.608 for all six items and 0.618 for the finally remaining five items. The country specific results are similar.

**Conclusions:**

The five items which remain after removing ‘assurance’, i.e. the SERVQUAL-MOD-5, constitute a short patient satisfaction index which can usefully be applied for different medical conditions and in different countries.

## 1 Introduction

The first outcome addressed by any health care is patients’ health. However, in addition to this, patient satisfaction is a further important outcome as this can affect the extent to which the patients adhere to their health care and/or to the health care providers. Moreover, it also has a value in itself. Hence, there are good reasons to design health care in such a way that patients are satisfied. With regard to this purpose, adequate questionnaires for assessing patient satisfaction are required. Ideally, these questionnaires should be indices in the sense of Streiner [1]. This means the individual questionnaire items should address those characteristics of the health care which can be assumed to affect satisfaction; and a total value reflecting patient satisfaction should be formed by aggregating the values for the individual items. Such indices of patient satisfaction not only make possible to estimate the level of satisfaction; they also provide starting points for improving satisfaction. To be specific, those characteristics which are perceived as least sufficient are the first candidates for modification.

For many research purposes patient satisfaction questionnaires are needed which go beyond the sole property of being a satisfaction index. One of these properties is that the patient satisfaction questionnaire is as universal as possible, i.e. that it can be applied to all kinds of care and all kinds of care providers and in all cultural contexts. Such a universal satisfaction questionnaire would make it possible to investigate cultural differences in valuing different aspects of care and such a universal questionnaire would make possible comparisons between different kinds of cares and different kinds of care providers in different cultural contexts. This, in turn, would enhance the possibility of learning between different settings. A further property which is essential in many research contexts is that the questionnaire is short. This distinctly enhances patients’ willingness to complete the questionnaire; especially when variables other than patient satisfaction are also being assessed.

There are numerous examples of questionnaires which constitute indices of patient satisfaction [2–29]. These indices themselves are quite diverse. Some address satisfaction with a very specific kind of care such as neonatal intensive care [15] or psychiatric care for outpatients [22]. Other indices have a broader scope such as satisfaction with inpatient care in general [9,16,18,28,29]. However, some of the instruments with a broader scope are designed for a specific cultural context [14,17,28] and there are only a few attempts for providing universal indices [3,23]. Yet, each of these indices has hitherto been psychometrically analysed in only one country. Moreover, research aimed at developing universal short indices for patient satisfaction is still in such an early state that further attempts might be fruitful, and this research might benefit from input from adjacent research areas.

One important adjacent research area is consumer research. This research has produced an instrument for assessing perceived service quality: SERVQUAL (SERVice QUALity) [30]. The original version of SERVQUAL consists of 22 items which all refer to different characteristics of service. In the standard application of SERVQUAL these items are presented twice. The study participants are first of all asked to rate the extent to which the different characteristics are relevant for the service in question. Subsequently, the study participants are asked to rate to which extent these characteristics actually hold true. In the SERVQUAL terminology, the first is referred to as ‘expectation’ and the second as ‘perception’. An aggregated measure which is meant to reflect perceived service quality is formed by adding the item specific differences between scores for expectations and perceptions. Originally, SERVQUAL was conceived for assessing perceived service quality in general rather than, specifically, perceived service quality of health care. Accordingly, the first services to which SERVQUAL has been applied were those of a bank, a credit card company, a repair and maintenance company, and a telephone company [30]. Only later was SERVQUAL applied to health care [31–42].

The approach of basing the aggregate value on differences between perceptions and expectations is specific for SERVQUAL. This approach is implied by the SERVQUAL developers’ understanding of perceived service quality. They consider this construct as something completely subjective and postulate that perceived service quality is high when perceptions are better than expectations and low in the opposite case [30]. There is, in fact, some justification for this theoretical conception. However, if one seeks objective features affecting satisfaction only the perceptions are relevant and not the expectations, so the perception module alone could potentially be used as a proper index of patient satisfaction. Hence, this module comes close to the short universal index of patient satisfaction envisaged here.

In its present form, however, the perception module of SERVQUAL still has two shortcomings: (1) it is too long; and (2) one feature, which has been shown to be essential for patient satisfaction, i.e. the care with which the personnel communicates with the patient [43], is not addressed by the present version of SERVQUAL. Hence, the universal short index envisaged here could perhaps be produced by selecting those of the 22 SERVQUAL items which are most important and by adding an item regarding the ‘carefulness of communication’. Such an approach is realized in the study presented here. The index resulting from this approach is subjected to psychometric analyses and further modified in reaction to the results of these analyses. The psychometric analyses are performed with data collected in a European project concerned with health provider networks [44]. In this project surveys with type 2 diabetes patients and with stroke patients were performed in England, Finland, Germany, Greece, the Netherlands and Spain and the items resulting from shortening the perception module of SERVQUAL and adding a communication item were included in the survey questionnaires. With these data the psychometric properties of the items cannot only be compared across different kinds of care but also across six different language versions and thereby, perhaps, six different cultural contexts.

## 2 Methods

### 2.1 The basic item set

The items selected from SERVQUAL were identified using the results of a principal component analysis reported by the SERVQUAL developers [30]. This principal component analysis produced five different components: ‘Tangibles’, ‘Reliability’, ‘Responsiveness’, ‘Assurance’ and ‘Empathy’ [30]. As the SERVQUAL items address possible causes of satisfaction and not its effects, the component structure is not implied by the construct measured, i.e. satisfaction, but by the characteristics of the services investigated. Correspondingly, the component structure cannot be seen as a characteristic of the measurement instrument and can, therefore, not be expected to be stable across different contexts [45–47]. However, those features which highly correlate for the services investigated in one study will presumably also correlate highly for different services. Hence, those SERVQUAL items which best reflect a component structure which has already been found are also likely to reflect the component structures in different contexts quite well. Accordingly, for each of the five components found by the SERVQUAL developers that item with the highest loading on this component was selected for the basic item set investigated here. The final basic item set resulted by adding an item addressing ‘carefulness of communication’ (see Table 1).

**Table 1:** The basic item set^a^.

English version	
Tangibles	The diabetes-related services have up-to-date equipment.
Reliability	The diabetes-related services provide their service at the time they promise to do so.
Responsiveness	Personnel of the diabetes-related services react promptly to my requests.
Assurance	Personnel of the diabetes-related services are polite.
Empathy	Personnel of the diabetes-related services give me personal attention.
Communication	Personnel of the diabetes-related services communicate carefully with me.
Answer categories^b^	Lower boundary: ‘Strongly disagree‘; upper boundary: ‘strongly agree’
Finnish version	
Tangibles	Käyttämistäni diabetekseen liittyvistä palveluista löytyy ajanmukaiset laitteet.
Reliability	Käyttämäni diabetekseen liittyvät palvelut palvelevat minua niin pian kuin lupaavatkin.
Responsiveness	Henkilökunta toteuttaa toiveeni nopeasti.
Assurance	Henkilökunta on kohteliasta.
Empathy	Saan henkilökunnalta henkilökohtaista huomiota.
Communication	Henkilökunta keskustelee kanssani ajatuksella.
Answer categories^b^	Lower boundary: ‘Täysin eri mieltä’; upper boundary ‘Täysin samaa mieltä’
German version	
Tangibles	Die auf den Diabetes bezogenen Dienste verfügen über eine moderne Ausstattung
Reliability	Die auf den Diabetes bezogenen Dienste erbringen ihre Leistungen zum versprochenen Zeitpunkt.
Responsiveness	Das Personal der auf den Diabetes bezogenen Dienste reagiert umgehend auf meine Wünsche.
Assurance	Das Personal der auf den Diabetes bezogenen Dienste ist höflich.
Empathy	Das Personal der auf den Diabetes bezogenen Dienste schenkt mir persönlich Aufmerksamkeit.
Communication	Das Personal der auf den Diabetes bezogenen Dienste kommuniziert sorgfältig mit mir.
Answer categories^b^	Lower boundary: ‘Stimme gar nicht zu‘; upper boundary: ‘Stimme voll zu’
Greek version	
Tangibles	Οι σχετικές με το διαβήτη υπηρεσίες έχουν σύγχρονο εξοπλισμό
Reliability	Οι σχετικές με το διαβήτη υπηρεσίες παρέχουν τις υπηρεσίες τους στο χρονικό διάστημα που υπόσχονται ότι θα το κάνουν
Responsiveness	Το προσωπικό των σχετικών με το διαβήτη υπηρεσιών ανταποκρίνεται άμεσα στα αιτήματά μου
Assurance	Το προσωπικό των σχετικών με το διαβήτη υπηρεσιών είναι ευγενικό
Empathy	Το προσωπικό των σχετικών με το διαβήτη υπηρεσιών με προσέχει
Communication	Το προσωπικό των σχετικών με το διαβήτη υπηρεσιών επικοινωνεί μαζί μου προσεκτικά
Answer categories^b^	Lower boundary: ‘Διαφωνώ πολύ‘; upper boundary: ‘Συμφωνώ πολύ’
Dutch version	
Tangibles	De diabetesgerelateerde zorgverleners beschikken over moderne apparatuur.
Reliability	De diabetesgerelateerde zorgverleners leveren hun diensten op het afgesproken tijdstip.
Responsiveness	De diabetesgerelateerde zorgverleners personeel reageren direct op mijn verzoeken.
Assurance	De diabetesgerelateerde zorgverleners personeel zijn beleefd.
Empathy	De diabetesgerelateerde zorgverleners personeel hebben persoonlijke aandacht voor me.
Communication	De diabetesgerelateerde zorgverleners personeel communiceren zorgvuldig met me.
Answer categories^b^	Lower boundary: ‘Helemaal niet mee eens‘; upper boundary: ‘Helemaal mee eens’
Spanish version	
Tangibles	Los servicios tenían al día los equipos e instalaciones
Reliability	Daban sus servicios con puntualidad
Responsiveness	Los profesionales de estos servicios reaccionaban de inmediato a mis necesidades.
Assurance	Los profesionales eran educados conmigo.
Empathy	Los profesionales daban una atención personalizada.
Communication	Los profesionales se comunicaban conmigo detenidamente.
Answer categories^b^	Lower boundary: ‘Totalmente en desacuerdo‘; upper boundary: ‘totalmente de acuerdo’

^a^ The table contains the specification of the items for diabetes-related services. When the items are referred to a different entity or to experiences in the past the items must be modified accordingly.

^b^ Seven answer categories are applied.

The basic item set was first formulated in English and then translated into the other five study languages. Following the rules of cultural adaptation the translations were performed in four steps: (1) two professional interpreters who were native speakers of the target language translated the English original independently of each other into the target language; (2) a member of the study team in the respective country discussed differences between the two translations with both interpreters and constructed one single version which could be approved by both interpreters; (3) a professional interpreter with English as their native language translated the resulting version back into English; (4) a member of the study team in the respective country discussed possible difference between the back translation and the original version with the back interpreter and, in case of essential differences, modified the target language version so that the back interpreter thought that his or her back translation for the modified version would have been close enough to the original version.

### 2.2 Study settings and study participants

The basic item set was applied in two different surveys, one with type 2 diabetes patients and one with stroke patients.

The diabetes survey was performed for six different networks of providers of type 2 diabetes care, one for each study country. These networks were: the London Borough of Tower Hamlets in England; the region of Keski-Suomi in Finland; the city and rural district of Bamberg in Germany; the regional unit of Herakleion on the island of Crete in Greece; the region Nieuwe Waterweg Noord en Delft Westland Oostland in the Netherlands; and Valencia-La Fe Health Department in Spain. In England seven general physician practices associated with the Tower Hamlets Primary Care Trust were investigated; in Finland the health centers of eight municipalities within Keski-Suomi; in Germany the practices of one general physician and one diabetologist in the city of Bamberg, and of two general physicians and one diabetologist in the rural district of Bamberg; in Greece, five different institutions providing outpatient care for diabetes; in the Netherlands, five general practitioner health centres; and, in Spain, one primary healthcare area[48].

The stroke survey was performed similarly for six different networks of providers of stroke care, one for each study country. The core or each of these networks was a hospital with a stroke unit. The investigated hospitals were the Brighton and Sussex University Hospitals in England, Keski-Suomi Central Hospital in Finland, the neurological hospital at the University Medical Center of Erlangen in Germany, the General Hospital of Athens ‘Alexandra’ in Greece, TweeSteden Ziekenhuis and St. Elisabeth Ziekenhuis in Tilburg, which are now merged into ElisabethTweesteden Ziekenhuis, in the Netherlands, and Valencia-La Fe Health Department in Spain.

Both surveys were performed with the assistance of the care providers investigated. These providers selected the patients to be approached for participation according to criteria defined by the researchers. Inclusion criteria for participants of the diabetes survey were 1) that they were being treated for type 2 diabetes by the health providers investigated in the project and 2) that they were at least 18 years old [48]. Inclusion criteria for participants of the stroke survey were 1) that they had been treated for stroke by the health providers investigated in the project in the year 2010 and 2) that they were at least 18 years old. The patients were contacted either by post or directly given the questionnaire when visiting their health care provider. The patients who participated in the survey completed their questionnaires on their own without any intervention by personnel from the service provider or research team. Depending on the most feasible method for the particular provider, the participants returned their completed questionnaires either by mail directly to the local project study centres, or to the care provider who then passed them on to the study centres. Data for the diabetes survey were collected between October 2011 and March 2012 [48], those for the stroke survey between September 2011 and February 2012.

### 2.3 Ethics statement

The English diabetes survey was approved by the NHS National Research Ethics Service. The English stroke survey was performed as part of a service development exercise and therefore did not require ethics committee approval. The Finnish surveys were approved by the Ethics Committee of the Central Finland Health Care District. The German surveys were approved by the Ethics Committee of the Medical Faculty of the Friedrich-Alexander University in Erlangen-Nürnberg. The Greek diabetes survey was approved by the Scientific Committee of the hospital in Herakleion and the Greek stroke survey by the Ethics Committee of the hospital Alexandra. The Dutch diabetes survey was approved by the board of directors of the Primary Care Group ZEL and the stroke survey by the Ethics Committee of the St. Elisabeth Hospital in Tilburg. The Spanish surveys were approved by the Hospital La Fe Ethical Committee.

Permission for use of data was received from the NHS National Research Ethics Service (statistical data and access of patient records through the clinicians of the local diabetes research network), the Ethics Committee of the Central Finland Health Care District (statistical data at aggregate level), the Ethics Committee of the Medical Faculty of the Friedrich- Alexander University in Erlangen-Nürnberg (statistical data at aggregate level), the Scientific Committee of the hospital in Herakleion (statistical data and access to patient records), the Ethics Committee of the hospital Alexandra (statistical data and access to patient records), the Scientific Council of the IPCI system of the department of Medical Information of the Erasmus Medical Centre (statistical data at aggregate level), and the Hospital La Fe Ethical Committee (statistical data at aggregate level).

### 2.4 The survey questionnaires

Both survey questionnaires contained the basic item set. In the diabetes survey the items referred to the type 2 diabetes-related services (see Table 1), in the stroke survey to the hospital in which the patients had been treated. Accordingly, in the stroke surveys the items were formulated in the past tense whereas they were formulated in present tense in the diabetes surveys. In addition to the basic item set both questionnaires contained several further questions (most of which are not relevant for the analyses presented here). Those questions which are relevant, in both questionnaires, are those addressing age, gender, educational attainment, mastery of the language in which the questionnaire was formulated and the ‘general satisfaction’ with the entity which was referred to by the basic item set.

Educational attainment was assessed by asking participants whether they had left school at the minimum school leaving age of their country. Those answering ‘yes’ were classified as having a lower level of educational attainment than those who answered ‘no’. Mastery of the questionnaire language was assessed via two questions. In the English version of the questionnaire the first question was ‘What is your first language?’ and the categories ‘English’ and ‘Other, please specify’ were given as answer options. The second question was ‘If English is not your first language, how well do you master it?’ with the answer options ‘Not at all’, ‘Poorly’, ‘Moderately’, ‘Well’ and ‘Perfectly’. In the other language versions the word ‘English’ was replaced with the word for the language in which the questionnaire was formulated [49]. ‘General satisfaction’ was assessed with one question. In the diabetes survey this question was: ‘How satisfied are you with the supply of diabetes-related services you have experienced?’. In the stroke survey it was: ‘How satisfied were you with the hospital in which you were treated because of your stroke?’. In both surveys a 7-categorical scale with the lowest category labelled by ‘Extremely dissatisfied’ and the highest category by ‘Extremely satisfied’ was provided for answering the question.

### 2.5 Statistical analyses

Not all study participants returning a questionnaire were included in the analyses. One exclusion criterion was that the questionnaire language was not the respondent’s first language and that the respondent mastered the questionnaire only moderately or worse. A further exclusion criterion was that data for the basic item set or for the ‘general satisfaction’ question were missing.

As a prerequisite for the statistical analyses the six basic items and the ‘general satisfaction’ item were coded numerically with -3 for the lowest category and +3 for the highest category. The six basic items were then aggregated into a sum score. To get a general impression of the study participants, descriptive statistics for age, gender, educational attainment, the six basic items, the sum scores for the six basic items and the ‘general satisfaction’ item were computed. These descriptive statistics were mean, standard deviation, minimum and maximum for age, the six basic items, the sum scores and the ‘general satisfaction’ item; and relative frequencies for gender and educational attainment. The analyses were performed for all relevant partitions of the sample, i.e. separately for each combination of medical condition and country, for each medical condition with countries pooled, for each country with medical conditions pooled and for the total sample with countries and medical conditions pooled.

Differences with regard to age, the six basic items, the sum scores and the ‘general satisfaction’ item were tested using t-tests when medical conditions were compared and using analyses of variance when countries were compared. Differences with regard to gender and educational attainment were tested using Fisher’s exact test when medical conditions were compared and chi-square tests for contingency tables when countries were compared. As the questionnaire items are bounded to both sides and as, therefore, violations of the normality assumption must be expected; differences with regard to the six basic items, the sum scores, and the ‘general satisfaction item were also tested with distribution-free tests. These were the Mann-Whitney-U-test for comparisons between medical conditions and the Kruskal-Wallis-test for comparisons between countries. By way of this 186 different significance tests were performed. However, this was only done in order to give an impression of the specific features of the study samples and not for substantiating any general statements about the six study countries or the two medical conditions. Therefore no control for multiple testing was performed.

The psychometric analyses performed here are strictly based on the idea that the items constitute an index, i.e. that the items describe causes and not effects of the variable to be measured. This implies that the correlational structure between the items is not determined by the variable to be measured. This, in turn, implies that this correlational structure must be expected to be different within different contexts and that, for this reason, neither this structure nor statistics based upon this structure can be interpreted as a feature of the measurement instrument [1,45-47]. For this reason several analyses which have previously often been performed with patient questionnaires are not adequate. This includes analyses with models of item-response-theory, as for example the Rasch-model, and attempts to estimate the sum score’s reliability using Cronbach’s alpha. Accordingly, such analyses were not performed here.

However, although the correlations between the individual items are not primarily determined by the quantity to be measured, they reflect nevertheless important aspects of the contexts in which the surveys were performed. Therefore, the inter-item correlations were computed for all relevant partitions of the sample. Differences between the corresponding variance-covariance-matrices of different medical conditions or, respectively, different countries were tested. This was performed by comparing the variance-covariance-matrices determined under the assumption that the matrices are equal for the different countries or, respectively, medical conditions with the empirically found variance-covariance-matrices using the chi-square test provided by the statistic package AMOS in SPSS.

In addition to the statistical test, a descriptive measure for the similarity between the item-inter-correlation-matrices was also determined. This measure was particularly developed for the analyses presented here and will be referred to as the Normed Euclidean Distance Coefficient (NEDC) in the following text. This measure is

$$NEDC=1-{\left(\frac{\sum_{i=1}^{m-1}\sum_{j>i}^{m}{\left(r_{ij1}-r_{ij2}\right)}^{2}}{m(m-1)/2}\right)}^{1/2}$$ (1)

with *m* the number of items, *r*_*ij*1_ the correlation between items *i* and *j* in matrix 1, and *r*_*ij*2_ the correlation between items *i* and *j* in matrix 2. Note that

${\left(\sum_{i=1}^{m-1}\sum_{j>i}^{m}{\left(r_{ij1}-r_{ij2}\right)}^{2}\right)}^{1/2}$ is the Euclidean distance between the upper right off-diagonal triangles of the two matrices, whereas (*m*(*m*−1)/2)^1/2^ is the Euclidean distance between the upper right off-diagonal triangles of two matrices of the same size with one matrix only containing zero correlations and the other only correlations equal to one. In other words, the term subtracted from one is equal to the Euclidean distance between the two investigated matrices standardized with regard to a reference distance. This reference distance, in turn, is equal to the Euclidean distance between a matrix with only zero correlations in the off-diagonal cells and a matrix with only correlations equal to one. Correspondingly, the *NEDC* is equal to one when both matrices to be compared are equal; on the other hand, the *NEDC* is equal to zero when the Euclidean distance between the two matrices equals the reference distance.

Matrices belonging to the two different medical conditions were directly compared using the *NEDC*. For matrices belonging to the six different study countries the means of the *NEDCs* determined over all 15 different pairs of countries were applied.

As a first step for testing the validity of the individual six basic items their correlations with ‘general satisfaction’ with the health care or, respectively, health care provider were computed. The ‘general satisfaction’ item addresses exactly that construct which is intended to be measured by the patient satisfaction index; however, it is presumed to be less reliable than the sum score because the sum score is based on several items. The correlations with ‘general satisfaction’ were computed for all relevant partitions of the sample.

As a second step for testing the validity of the individual items, cumulative logistic regression analyses with the items as predictors and ‘general satisfaction’ as the criterion with enforced equal distance between the categories were computed. Cumulative logistic regression rather than linear regression was applied because the basic assumptions of the linear regression model are necessarily violated when the criterion variable is bounded to both sides (as holds true for the ‘general satisfaction’ item). The regression analyses were performed separately for each combination of medical condition and country, for each medical condition with countries pooled, for each country with medical conditions pooled and for the total sample with countries and medical conditions pooled. Study participants with the same medical condition or from the same country might be more similar to each other than participants with different medical conditions or from different countries., For this reason, descriptive and inferential statistics might be distorted. To cope with this possibility, dummy variables for each combination of medical condition and country (except for one reference combination) were added when more than one combination was considered in the same analysis. Where an item was consistently shown to have a statistically significant negative contribution to the prediction of ‘general satisfaction’ then this item was removed from the item set. The multivariate analyses just described were then repeated with the remaining items.

For the final item set differences between regression coefficients from different countries or medical conditions were also tested. For this purpose, regression analyses with interaction terms between items and countries or respectively medical conditions were computed and compared with regression analyses without such interaction terms. A statistically significant decrease of deviance due to adding the interaction terms was interpreted as evidence for differences between the regression coefficients belonging to different countries or respectively different medical conditions. Moreover, to judge the extent to which the SERVQUAL-items predict general satisfaction, a specific kind of Nagelskerke’s pseudo R-square was computed for each partition of the data. The specific characteristic of these R-squares was their basis model, i.e. the model with which the regression model is compared. Usually, the predictions of the regression model are only compared with the relative frequency of the criterion in the total sample. Instead, in the analyses presented here, the model including the SERVQUAL-items was compared a model without the SERVQUAL-items but with all further predictor variables included in the model with the SERVQUAL-items.

The validity of the sum scores of all items sets emerging in the process just described was also tested. This was performed via the correlations with the item addressing ‘general satisfaction’. These correlations were computed for all relevant partitions of the sample.

## 3 Results

In the diabetes survey, 6245 questionnaires were distributed of which 1638 were returned and 1202 met the inclusion criteria (see Table 2). The proportion of excluded questionnaires was largest in England (48.0%) which was due to the fact that about 40% of all respondents in this sample were of Bangladeshi ethnicity who, due to lower levels of stated proficiency in the English language, did not meet the inclusion criteria for this analysis. Altogether, 19.2% of the questionnaires distributed in the diabetes survey were included in the final analyses with the inclusion proportions varying from 7.4% for England to 50.0% for Germany. In the stroke survey, 2369 questionnaires were distributed of which 826 were returned and 682 met the inclusion criteria (see Table 2). In the stroke survey nearly all respondents had sufficient proficiency in the questionnaire language so that only a very few respondents had to be excluded due to insufficient proficiency. Altogether, 28.8% of the questionnaires distributed in the stroke survey were included in the final analyses with the proportions of the inclusion proportions ranging from 23.2% for Finland to 46.0% for Greece. For both surveys together the proportion of finally included questionnaires in relation to the questionnaires distributed is 21.9% (see Table 2).

**Table 2:** Information about the emergence of the sample^a^.

	Question-naires distributed	Questionnaires returned	Sufficient language competence	Sufficient data^b^	Participants included
Diabetes survey					
England	3343	475 (14.2%)	313 (9.4%)	373 (11.2%)	247 (7.4%)
Finland	436	183 (42.0%)	183 (42.0%)	160 (36.7%)	160 (36.7%)
Germany	462	286 (61.9%)	282 (61.0%)	235 (50.9%)	231 (50.0%)
Greece	600	179 (29.8%)	179 (29.8%)	152 (25.3%)	152 (25.3%)
The Netherlands	779	400 (51.3%)	387 (49.7%)	326 (41.8%)	316 (40.6%)
Spain	625	115 (18.4%)	115 (18.4%)	96 (15.4%)	96 (15.4%)
All countries	6245	1638 (26.2%)	1459 (23.4%)	1342 (21.5%)	1202 (19.2%)
Stroke survey					
England	346	120 (34.7%)	119 (34.4%)	102 (29.5%)	101 (29.2%)
Finland	600	190 (31.7%)	189 (31.5%)	139 (23.2%)	139 (23.2%)
Germany	366	126 (34.4%)	123 (33.6%)	110 (30.1%)	107 (29.2%)
Greece	126	65 (51.6%)	65 (51.6%)	58 (46.0%)	58 (46.0%)
The Netherlands	625	224 (35.8%)	223 (35.7%)	186 (29.8%)	185 (29.6%)
Spain	306	101 (33.0%)	100 (32.7%)	93 (30.4%)	92 (30.1%)
All countries	2369	826 (34.9%)	819 (34.6%)	688 (29.0%)	682 (28.8%)
Both surveys together					
England	3689	595 (16.1%)	432 (11.7%)	475 (12.9%)	348 (9.4%)
Finland	1036	373 (36.0%)	372 (35.9%)	299 (28.9%)	299 (28.9%)
Germany	828	412 (49.8%)	405 (48.9%)	345 (41.7%)	338 (40.8%)
Greece	726	244 (33.6%)	244 (33.6%)	210 (28.9%)	210 (28.9%)
The Netherlands	1404	624 (44.4%)	610 (43.4%)	512 (36.5%)	501 (35.7%)
Spain	931	216 (23.2%)	215 (23.1%)	189 (20.3%)	188 (20.2%)
All countries	8614	2464 (28.6%)	2278 (26.4%)	2030 (23.6%)	1884 (21.9%)

^a^ Percentages in brackets refer to the number of questionnaires distributed.

^b^ Participants who have provided data for all items of the SERVQUAL-MOD-6 and for the ‘general satisfaction’ question.

The respondents tended to be older with the age mean of the total sample being 66.6. The majority was male and higher educated (see Table 3). Educational attainment differs essentially between the countries both for the two medical conditions separately and for the total sample. There is also a statistically significant effect between the countries with regard to age within the two medical condition specific sub-samples but these effects level out in the total sample. The two medical condition specific sub-samples differ distinctly with regard to age with the members of the stroke sub-sample being older than those of the diabetes sub-sample (see Table 3). The average values for the six basic items, the corresponding sum score, and the ‘general satisfaction’ are all in the positive half of the measurement range (see Table 4). The two significance tests which have both been applied for testing the same differences, i.e. a test presupposing a normal distribution and a distribution-free test, mostly yield the same results. Most of the differences between the countries and several of the differences between the medical conditions are statistically significant (see Table 4).

**Table 3:** Socio-demographic characteristics^a^.

Country	Characteristics	Diabetes survey	Stroke survey	Both surveys	Comparisons^b^
England	Age in years	63.2 (12.5); 28–89; (241)	74.3 (10.7); 44–93; (99)	66.4 (13.0); 28–93; (340)	***
	Male gender	62.6%; (238)	64.3%; (98)	63.1%; (336)	---
	High education	37.3%; (217)	50.5%; (93)	41.3%; (310)	*
Finland	Age in years	64.1 (9.8); 34–98; (157)	69.0 (12.8); 30–91; (134)	66.3 (11.5); 30–98; (291)	***
	Male gender	63.2%; (155)	51.5%; (130)	57.9%; (285)	---
	High education	58.8%; (148)	65.0%; (120)	61.6%; (268)	---
Germany	Age in years	65.4 (11.3); 21–90; (227)	66.9 (13.6); 21–90; (100)	65.9 (12.1); 21–90; (327)	---
	Male gender	49.6%; (226)	58.6%; (99)	52.3%; (325)	---
	High education	66.7%; (219)	74.7%; (95)	69.1%; (314)	---
Greece	Age in years	65.8 (10.7); 30–89; (151)	72.8 (11.0); 43–97; (57)	67.8 (11.2); 30–97; (208)	***
	Male gender	58.3%; (151)	50.0%; (58)	56.0%; (209)	---
	High education	25.0%; (148)	45.3%; (53)	30.3%; (201)	**
The Netherlands	Age in years	64.9 (10.3); 29–89; (311)	69.5 (12.8); 26–99; (183)	66.6 (11.5); 26–99; (494)	**
	Male gender	58.6%; (304)	62.1%; (182)	59.9%; (486)	---
	High education	76.7%; (300)	62.6%; (163)	71.7%; (463)	**
Spain	Age in years	67.9 (12.1); 30–92; (91)	66.5 (12.4); 29–85; (90)	67.2 (12.2); 29–92; (181)	---
	Male gender	57.8%; (90)	66.3%; (89)	62.0%; (179)	---
	High education	30.2%; (86)	31.0%; (84)	30.6%; (170)	---
All countries	Age in years	64.9 (11.1); 21–98; (1178)	69.6 (12.7); 21–99; (663)	66.6 (11.9); 21–99; (1841)	***
	Male gender	58.2%; (1164)	59.3%; (656)	58.6%; (1820)	---
	High education	54.3%; (1118)	57.2%; (608)	55.3%; (1726)	---
Comparisons^c^	Age in years	**	***	---	
	Male gender	---	---	---	
	High education	***	***	***	

^a^ Due to missing values the statistics for social demographic characteristics are often based on fewer participants than the participants included. The cell entries are ‘Mean (Standard deviation); Minimum-Maximum; (sample size)’ for age in years and ‘Percentage; (sample size)’ for male gender and higher education. Symbols mean ‘---‘ = not significant

‘*’ = p<0.05

‘**’ = p<0.01

‘***’ = p<0.001.

^b^ Difference between medical conditions: two-tailed t-test for independent samples with unequal variances for age; Fisher’s exact test for contingency table for gender and education.

^c^ Difference between countries: analysis of variance for age; chi-square test for contingency tables for gender and education.

**Table 4:** Basic items, sum of basic items and ‘general satisfaction’^a^.

Variable	Diabetes survey	Stroke survey	Both surveys	Comparisons^b^
England				
Tangibles	1.6 (1.5)	1.9 (1.4)	1.7 (1.5)	--- (---)
Reliability	1.8 (1.5)	1.7 (1.7)	1.8 (1.6)	--- (---)
Responsiveness	1.7 (1.6)	1.5 (1.8)	1.6 (1.7)	--- (---)
Assurance	2.2 (1.5)	2.2 (1.4)	2.2 (1.4)	--- (---)
Empathy	2.0 (1.5)	1.8 (1.7)	1.9 (1.5)	--- (---)
Communication	1.9 (1.5)	1.7 (1.8)	1.9 (1.6)	--- (---)
Sum of basic items	11.1 (8.3)	10.8 (8.4)	11.0 (8.3)	--- (---)
Satisfaction	1.7 (1.5)	1.7 (1.6)	1.7 (1.5)	--- (---)
Finland				
Tangibles	2.2 (1.3)	2.2 (1.3)	2.2 (1.3)	--- (---)
Reliability	2.2 (1.5)	2.0 (1.5)	2.1 (1.5)	--- (---)
Responsiveness	2.2 (1.3)	1.9 (1.5)	2.0 (1.4)	--- (---)
Assurance	2.5 (1.0)	2.4 (1.2)	2.5 (1.1)	--- (---)
Empathy	2.1 (1.4)	1.9 (1.6)	2.0 (1.5)	--- (---)
Communication	2.2 (1.3)	1.6 (1.8)	1.9 (1.6)	** (*)
Sum of basic items	13.3 (6.9)	12.0 (7.4)	12.7 (7.2)	--- (---)
Satisfaction	2.2 (1.1)	1.9 (1.4)	2.1 (1.3)	--- (---)
Germany				
Tangibles	2.2 (1.2)	2.5 (1.3)	2.3 (1.2)	--- (***)
Reliability	2.3 (1.3)	2.4 (1.4)	2.3 (1.3)	--- (*)
Responsiveness	2.4 (1.2)	2.0 (1.6)	2.2 (1.4)	* (*)
Assurance	2.6 (0.9)	2.3 (1.6)	2.5 (1.2)	* (---)
Empathy	2.5 (1.1)	1.8 (1.7)	2.3 (1.3)	*** (***)
Communication	2.5 (1.1)	1.9 (1.6)	2.3 (1.3)	*** (**)
Sum of basic items	14.4 (6.0)	12.9 (8.2)	14.0 (6.8)	--- (---)
Satisfaction	1.8 (1.4)	1.9 (1.4)	1.9 (1.4)	--- (---)
Greece				
Tangibles	0.9 (2.0)	1.6 (1.7)	1.1 (1.9)	* (*)
Reliability	1.2 (1.8)	2.0 (1.5)	1.4 (1.8)	*** (***)
Responsiveness	1.4 (1.8)	2.4 (1.0)	1.7 (1.7)	*** (***)
Assurance	2.0 (1.6)	2.6 (0.7)	2.2 (1.5)	*** (*)
Empathy	2.0 (1.6)	2.6 (0.8)	2.1 (1.4)	*** (*)
Communication	1.3 (2.0)	2.4 (1.1)	1.6 (1.9)	*** (***)
Sum of basic items	8.7 (8.8)	13.6 (5.6)	10.1 (8.3)	*** (***)
Satisfaction	1.2 (1.6)	1.9 (1.4)	1.4 (1.6)	*** (***)
The Netherlands				
Tangibles	1.7 (1.5)	2.3 (1.4)	1.9 (1.5)	*** (***)
Reliability	2.3 (1.3)	2.2 (1.4)	2.3 (1.3)	--- (---)
Responsiveness	2.3 (1.3)	2.0 (1.5)	2.2 (1.4)	--- (*)
Assurance	2.6 (1.2)	2.4 (1.3)	2.5 (1.2)	--- (*)
Empathy	2.5 (1.2)	2.2 (1.5)	2.4 (1.3)	* (***)
Communication	2.4 (1.3)	1.9 (1.6)	2.2 (1.4)	*** (***)
Sum of basic items	13.8 (7.0)	13.1 (7.8)	13.6 (7.3)	--- (---)
Satisfaction	2.3 (1.1)	2.0 (1.5)	2.2 (1.3)	--- (---)
Spain				
Tangibles	1.5 (1.8)	2.0 (1.6)	1.8 (1.7)	* (**)
Reliability	1.1 (2.0)	1.9 (1.8)	1.5 (2.0)	** (***)
Responsiveness	1.5 (1.8)	1.9 (1.9)	1.7 (1.8)	--- (---)
Assurance	2.3 (1.5)	2.2 (1.7)	2.2 (1.6)	--- (---)
Empathy	1.8 (1.8)	1.9 (1.9)	1.9 (1.9)	--- (---)
Communication	1.7 (2.0)	2.0 (1.9)	1.8 (1.9)	--- (---)
Sum of basic items	9.8 (9.5)	11.9 (9.5)	10.8 (9.5)	--- (*)
Satisfaction	1.3 (1.6)	2.2 (1.3)	1.7 (1.5)	*** (***)
All countries				
Tangibles	1.7 (1.6)	2.2 (1.4)	1.9 (1.5)	*** (***)
Reliability	1.9 (1.6)	2.1 (1.6)	2.0 (1.6)	--- (**)
Responsiveness	2.0 (1.5)	1.9 (1.6)	2.0 (1.6)	--- (---)
Assurance	2.4 (1.3)	2.3 (1.4)	2.4 (1.3)	--- (---)
Empathy	2.2 (1.4)	2.0 (1.6)	2.1 (1.5)	** (**)
Communication	2.1 (1.5)	1.9 (1.7)	2.0 (1.6)	** (*)
Sum of basic items	12.3 (7.8)	12.4 (8.0)	12.4 (7.9)	--- (---)
Satisfaction	1.9 (1.4)	2.0 (1.4)	1.9 (1.4)	--- (*)
Comparisons^c^				
Tangibles	*** (***)	*** (***)	*** (***)	
Reliability	*** (***)	* (***)	*** (***)	
Responsiveness	*** (***)	* (*)	*** (***)	
Assurance	*** (***)	--- (---)	*** (***)	
Empathy	*** (***)	* (**)	*** (***)	
Communication	*** (***)	* (*)	*** (***)	
Sum of basic items	*** (***)	--- (*)	*** (***)	
Satisfaction	*** (***)	--- (*)	*** (***)	

^a^ The cell entries are ‘Mean (Standard deviation. All items are coded from -3 for ‘Strongly disagree‘ or, respectively, ‘Extremely dissatisfied’ to 3 for ‘Strongly agree’ or, respectively, ‘Extremely satisfied’. Accordingly, the possible values for the sum of the basic items range from -18 to 18. Symbols mean ‘---‘ = not significant

‘*’ = p<0.05

‘**’ = p<0.01

‘***’ = p<0.001. As there are no missing values for the basic items and the ‘general satisfaction’ item the sizes for all sub-samples are equal to the corresponding numbers in Table 2.

^b^ Differences between medical conditions: two-tailed t-tests for independent samples with unequal variances (two-tailed Mann-Whitney-U-test).

^c^ Differences between countries: analyses of variance (Kruskal-Wallis-test).

All basic six items correlate positively with each other in all investigated partitions of the data set (see Table 5). With one exception, i.e. the correlation between tangibles and assurance in the Greek stroke survey, the deviation from zero is statistically significant for all correlations. All investigated differences between variance-covariance-matrices belonging to the item-inter-correlation-matrices are statistically significant (see Table 5). In spite of these statistically significant differences, the *NEDCs* show much similarity between the item-inter-correlation-matrices. This similarity, however, is higher between matrices belonging to different medical conditions than between matrices belonging to different countries.

**Table 5:** Correlations between the 6 basic items^a^.

Predictors	Diabetes survey					Stroke survey					Both surveys					Comparison^b^
England																
	Rel.	Res.	Ass.	Emp.	Com.	Rel.	Res.	Ass.	Emp.	Com.	Rel.	Res.	Ass.	Emp.	Com.	*** NEDC = 0.87
Tangibles	0.80	0.78	0.68	0.71	0.71	0.71	0.56	0.48	0.57	0.51	0.76	0.71	0.62	0.66	0.64	
Reliability		0.86	0.70	0.82	0.77		0.72	0.68	0.70	0.71		0.81	0.69	0.78	0.75	
Responsiveness			0.78	0.85	0.85			0.62	0.79	0.77			0.73	0.83	0.82	
Assurance				0.86	0.81				0.76	0.75				0.83	0.78	
Empathy					0.88					0.93					0.90	
Finland																
	Rel.	Res.	Ass.	Emp.	Com.	Rel.	Res.	Ass.	Emp.	Com.	Rel.	Res.	Ass.	Emp.	Com.	*** NEDC = 0.87
Tangibles	0.85	0.71	0.68	0.70	0.72	0.64	0.64	0.54	0.48	0.52	0.75	0.67	0.60	0.58	0.60	
Reliability		0.85	0.69	0.78	0.79		0.73	0.58	0.64	0.69		0.79	0.64	0.71	0.72	
Responsiveness			0.73	0.68	0.77			0.66	0.62	0.65			0.70	0.65	0.70	
Assurance				0.66	0.74				0.66	0.60				0.66	0.65	
Empathy					0.83					0.73					0.77	
Germany																
	Rel.	Res.	Ass.	Emp.	Com.	Rel.	Res.	Ass.	Emp.	Com.	Rel.	Res.	Ass.	Emp.	Com.	*** NEDC = 0.94
Tangibles	0.75	0.65	0.63	0.67	0.67	0.78	0.59	0.71	0.62	0.58	0.76	0.60	0.63	0.59	0.58	
Reliability		0.68	0.70	0.70	0.69		0.74	0.76	0.73	0.73		0.68	0.70	0.67	0.67	
Responsiveness			0.75	0.80	0.79			0.83	0.88	0.90			0.79	0.84	0.85	
Assurance				0.82	0.79				0.80	0.82				0.81	0.81	
Empathy					0.86					0.88					0.88	
Greece																
	Rel.	Res.	Ass.	Emp.	Com.	Rel.	Res.	Ass.	Emp.	Com.	Rel.	Res.	Ass.	Emp.	Com.	*** NEDC = 0.91
Tangibles	0.69	0.56	0.35	0.33	0.44	0.75	0.54	0.26	0.42	0.44	0.71	0.57	0.35	0.35	0.45	
Reliability		0.77	0.51	0.48	0.52		0.68	0.40	0.59	0.61		0.76	0.50	0.51	0.55	
Responsiveness			0.63	0.64	0.67			0.57	0.68	0.68			0.64	0.66	0.69	
Assurance				0.84	0.65				0.86	0.64				0.85	0.67	
Empathy					0.69					0.91					0.72	
The Netherlands																
	Rel.	Res.	Ass.	Emp.	Com.	Rel.	Res.	Ass.	Emp.	Com.	Rel.	Res.	Ass.	Emp.	Com.	*** NEDC = 0.92
Tangibles	0.61	0.69	0.57	0.63	0.63	0.76	0.70	0.73	0.68	0.61	0.64	0.66	0.61	0.61	0.57	
Reliability		0.82	0.80	0.81	0.81		0.84	0.80	0.73	0.74		0.83	0.80	0.78	0.78	
Responsiveness			0.80	0.86	0.85			0.83	0.82	0.83			0.81	0.84	0.84	
Assurance				0.92	0.89				0.83	0.75				0.88	0.82	
Empathy					0.96					0.89					0.92	
Spain																
	Rel.	Res.	Ass.	Emp.	Com.	Rel.	Res.	Ass.	Emp.	Com.	Rel.	Res.	Ass.	Emp.	Com.	*** NEDC = 0.90
Tangibles	0.70	0.76	0.70	0.73	0.64	0.79	0.64	0.63	0.58	0.60	0.75	0.71	0.65	0.66	0.62	
Reliability		0.81	0.60	0.63	0.56		0.83	0.71	0.64	0.79		0.81	0.63	0.63	0.66	
Responsiveness			0.73	0.82	0.74			0.75	0.69	0.80			0.73	0.75	0.77	
Assurance				0.80	0.68				0.88	0.76				0.84	0.72	
Empathy					0.83					0.75					0.79	
All countries																
	Rel.	Res.	Ass.	Emp.	Com.	Rel.	Res.	Ass.	Emp.	Com.	Rel.	Res.	Ass.	Emp.	Com.	*** NEDC = 0.95
Tangibles	0.72	0.70	0.59	0.61	0.63	0.73	0.60	0.58	0.54	0.53	0.72	0.65	0.58	0.57	0.58	
Reliability		0.81	0.67	0.71	0.70		0.76	0.68	0.67	0.71		0.79	0.67	0.69	0.70	
Responsiveness			0.75	0.79	0.79			0.74	0.76	0.78			0.74	0.77	0.79	
Assurance				0.83	0.77				0.79	0.72				0.82	0.75	
Empathy					0.84					0.83					0.84	
Comparison^c^	*** ; Mean NEDC = 0.87					*** ; Mean NEDC = 0.87					*** ; Mean NEDC = 0.89					

^a^ For sample sizes see Table 2. The sub-titles for ‘Diabetes survey’, ‘Stroke survey’, and ‘Both surveys’ are ‘Rel.’ = ‘Reliability’, ‘Res.’ = ‘Responsiveness’, ‘Ass.’ = ‘Assurance’, ‘Emp.’ = ‘Empathy’, and ‘Com.’ = ‘Communication’. Symbols mean ‘---‘ = not significant

‘*’ = p<0.05

‘**’ = p<0.01

‘***’ = p<0.001.

^b^ Comparison of variance-covariance-matrices for medical conditions. Cell entries: significance level for chi-square test for equality for variance-covariance matrices (21 degrees of freedom); Normed Euclidean Distance Coefficient (see Formula 1).

^c^ Comparison of variance-covariance-matrices for countries. Cell entries: significance level for chi-square test for equality for variance-covariance matrices (129 degrees of freedom); mean of NEDCs (see Formula 1) for all 15 different pairs of countries.

In all partitions of data, all items correlate positively with ‘general satisfaction’. With two exceptions, the deviations of these correlations from zero are statistically significant. The two exceptions are the correlations of ‘general satisfaction’ with tangibles and with assurance both in the stroke survey in Spain. In the total sample, the correlations are 0.48 for tangibles, 0.56 for reliability, 0.58 for responsiveness, 0.47 for assurance, 0.53 for empathy, and 0.56 for communication.

In the regression analysis performed for the total sample with ‘general satisfaction’ as criterion and the six basic items as predictors the regression coefficients are 0.143 for tangibles, 0.183 for reliability, 0.319 for responsiveness, -0.209 for assurance, 0.208 for empathy, and 0.257 for communication. For all coefficients, the deviations from zero are statistically significant. This means that five of the six items actually contribute positively to the prediction of satisfaction, but one, i.e. assurance, contributes negatively. This effect also exists in both medical condition specific analyses with all countries pooled and in three of the six country specific analyses with medical conditions pooled. For the other three countries, there is no statistically significant effect, but a negative tendency for the assurance item. The assurance item also contributes negatively to the prediction of ‘general satisfaction’ in seven of the 12 regression analyses performed for the individual combinations of medical condition and country. In six of seven cases this contribution is statistically significant whereas there is no statistically significant effect for the five analyses in which assurance contributes positively to predicting ‘general satisfaction’.

Following the results just described, the assurance item was removed from the item set and the regression analyses were repeated with the remaining five items. In the analysis for the total sample, the regression coefficients of all five items are positive and their deviation from zero is statistically significant (see Table 6). There are strong differences between the regression coefficients obtained for the different countries and slight differences between the coefficients obtained for the different medical conditions. With one exception, i.e. the differences associated with medical conditions in England, all differences are statistically significant (see Table 6). Eleven of the 60 regression coefficients computed for the individual combinations of medical condition and country are negative and, in three of these cases, the deviation from zero is statistically significant. However, the negative coefficients are distributed over four of the five items with communication being the exception (see Table 6). Hence, there seems to be no need for removing a further item.

**Table 6:** Regression of ‘general satisfaction’ on the final 5 SERVQUAL items^a^.

Predictors	Diabetes survey	Stroke survey	Both surveys	Comparison^b^
England				
Tangibles	0.246 (0.100); 1.279; *	-0.158 (0.152); 0.853; ---	0.115 (0.080); 1.122; ---	0.002; ---
Reliability	0.352 (0.130); 1.423; **	0.543 (0.150); 1.720; ***	0.405 (0.093); 1.499; ***	
Responsiveness	0.451 (0.131); 1.570; ***	0.470 (0.137); 1.600; ***	0.475 (0.093); 1.609; ***	
Empathy	0.150 (0.144); 1.162; ---	0.295 (0.234); 1.343; ---	0.154 (0.118); 1.166; ---	
Communication	0.265 (0.130); 1.304; *	0.174 (0.211); 1.190; ---	0.284 (0.106); 1.328; **	
Nagelkerke’s Pseudo R^2^	0.490; ***	0.513; ***	0.493; ***	
Finland				
Tangibles	0.213 (0.185); 1.237; ---	0.301 (0.115); 1.351; **	0.320 (0.093); 1.377; ***	0.013; **
Reliability	1.180 (0.255); 3.255; ***	0.066 (0.114); 1.069; ---	0.292 (0.102); 1.339; **	
Responsiveness	-0.199 (0.191); 0.820; ---	0.267 (0.115); 1.306; *	0.189 (0.093); 1.209; *	
Empathy	-0.188 (0.222); 0.828; ---	0.225 (0.105); 1.252; *	0.158 (0.091); 1.171; ---	
Communication	0.184 (0.217); 1.202; ---	0.316 (0.100); 1.371; **	0.247 (0.090); 1.280; **	
Nagelkerke’s Pseudo R^2^	0.409; ***	0.369; ***	0.361; ***	
Germany				
Tangibles	0.196 (0.107); 1.216; ---	0.690 (0.154); 1.993; ***	0.356 (0.084); 1.428; ***	0.009; **
Reliability	0.164 (0.103); 1.178; ---	-0.480 (0.164); 0.619; **	-0.027 (0.087); 0.973; ---	
Responsiveness	0.514 (0.118); 1.673; ***	0.155 (0.192); 1.167; ---	0.408 (0.100); 1.504; ***	
Empathy	0.009 (0.156); 1.009; ---	0.158 (0.181); 1.171; ---	0.059 (0.118); 1.061; ---	
Communication	0.036 (0.152); 1.036; ---	0.389 (0.194); 1.476; *	0.101 (0.117); 1.106; ---	
Nagelkerke’s Pseudo R^2^	0.205; ***	0.294; ***	0.220; ***	
Greece				
Tangibles	0.514 (0.076); 1.672; ***	0.241 (0.181); 1.273; ---	0.474 (0.068); 1.606; ***	0.018; ***
Reliability	-0.195 (0.100); 0.823; ---	0.401 (0.230); 1.493; ---	-0.046 (0.087); 0.955; ---	
Responsiveness	0.318 (0.103); 1.374; **	0.499 (0.290); 1.647; ---	0.254 (0.092); 1.290; **	
Empathy	0.280 (0.092); 1.323; **	-0.064 (0.616); 0.938; ---	0.298 (0.091); 1.347; **	
Communication	0.139 (0.075); 1.149; ---	1.085 (0.511); 2.959; *	0.192 (0.073); 1.212; **	
Nagelkerke’s Pseudo R^2^	0.371; ***	0.517; ***	0.375; ***	
The Netherlands				
Tangibles	0.036 (0.080); 1.037; ---	-0.504 (0.119); 0.604; ***	-0.134 (0.064); 0.875; *	0.010; ***
Reliability	0.124 (0.116); 1.133; .283	0.390 (0.124); 1.477; **	0.145 (0.079); 1.156; ---	
Responsiveness	0.498 (0.133); 1.645; ***	0.204 (0.129); 1.227; ---	0.394 (0.091); 1.483; ***	
Empathy	-0.059 (0.217); 0.943; ---	0.122 (0.142); 1.129; ---	-0.088 (0.112); 0.916; ---	
Communication	0.293 (0.212); 1.341; ---	0.351 (0.127); 1.420; **	0.413 (0.106); 1.511; ***	
Nagelkerke’s Pseudo R^2^	0.242; ***	0.250; ***	0.227; ***	
Spain				
Tangibles	-0.200 (0.128); 0.819; ---	-0.504 (0.119); 0.604; ***	-0.203 (0.088); 0.816; *	0.025; ***
Reliability	0.386 (0.110); 1.470; ***	0.390 (0.124); 1.477; **	0.208 (0.085); 1.231; *	
Responsiveness	-0.226 (0.164); 0.798; ---	0.204 (0.129); 1.227; ---	0.171 (0.104); 1.187; ---	
Empathy	0.579 (0.153); 1.784; ***	0.122 (0.142); 1.129; ---	0.129 (0.087); 1.138; ---	
Communication	0.187 (0.112); 1.206; ---	0.351 (0.127); 1.420; **	0.213 (0.081); 1.238; **	
Nagelkerke’s Pseudo R^2^	0.308; ***	0.152; ***	0.198; ***	
All countries				
Tangibles	0.225 (0.037); 1.252; ***	-0.012 (0.050); 0.988; ---	0.130 (0.029); 1.139; ***	0.003; ***
Reliability	0.163 (0.045); 1.177; ***	0.180 (0.055); 1.198; ***	0.171 (0.034); 1.186; ***	
Responsiveness	0.294 (0.050); 1.342; ***	0.268 (0.054); 1.308; ***	0.289 (0.037); 1.335; ***	
Empathy	0.184 (0.052); 1.202; ***	0.015 (0.060); 1.016; ---	0.103 (0.039); 1.108; **	
Communication	0.161 (0.048); 1.175; ***	0.335 (0.058); 1.398; ***	0.241 (0.036); 1.273; ***	
Nagelkerke’s Pseudo R^2^	0.306: ***	0.274; ***	0.289; ***	
Comparison^c^	0.016; ***	0.041; ***	0.016; ***	

^a^ For sample sizes see Table 2. Entries of regular cells: regression coefficient with criterion and all predictors coded from -3 to 3 (standard error of coefficient); odds ratio for the criterion variable increasing one unit when the corresponding predictor variable increases one unit; test for deviation of regression coefficient from zero. Entries for cells for Nagelkerke’s Pseudo R^2^: Nagelkerke’s Pseudo R^2^ with a model containing all predictors except the SERVQUAL item as basis model; significance level for deviation of coefficient from zero. Symbols are ‘---‘ = not significant

‘*’ = p<0.05

‘**’ = p<0.01

‘***’ = p<0.001.

^b^ Difference between medical conditions tested by comparing the model for both surveys together with a model with medical condition specific parametrization; entries are: the difference of Nagelkerke’s Pseudo R^2^ of the model with medical condition specific parametrization and the model for both surveys together; significance level for difference.

^c^ Difference between countries tested by comparing the model for all countries together with a model with country specific parametrization; entries are: the difference of Nagelkerke’s Pseudo R2 of the model with medical condition specific parametrization and the model for both surveys together; significance level for difference.

In the total sample the correlation between the sum score of the six basic items and the ‘general satisfaction’ is 0.608. The correlations for the individual combinations of country and medical condition range from 0.303 for the stroke survey in Spain to 0.787 for the stroke survey in Greece (see Table 7). After removing the assurance item, the correlations for the sum scores for the remaining five items increase in all partitions of the data except for the diabetes survey in Spain and the stroke surveys in England and Germany. In the latter four cases, the decrease is very small. In the total sample the correlation between the sum of the five included items and ‘general satisfaction’ increases to 0.618 (see Table 7).

**Table 7:** Correlations between sum scores and ‘general satisfaction’.

Country	Diabetes survey	Stroke survey	Both surveys
Sum score for all six items			
England	0.766	0.774	0.769
Finland	0.668	0.672	0.670
Germany	0.498	0.569	0.516
Greece	0.641	0.787	0.679
The Netherlands	0.559	0.462	0.511
Spain	0.579	0.303	0.467
All countries	0.638	0.561	0.608
Sum score for the remaining five items with assurance removed			
England	0.777	0.773	0.776
Finland	0.696	0.673	0.684
Germany	0.507	0.566	0.522
Greece	0.646	0.790	0.686
The Netherlands	0.564	0.472	0.519
Spain	0.578	0.323	0.483
All countries	0.646	0.570	0.618

## 4 Discussion

### 4.1 Assets and limitations of the study

The study presented here has both certain assets and limitations. An important asset is that the study has been conducted with regard to the care for two different medical conditions and in six different countries. Such a study design provides evidence as to how the results differ between different contexts and, thereby, to which extent they can be generalised. Hitherto no study has been published in which a patient satisfaction questionnaire has been investigated with a comparable study design. Hence, the study presented here not only provides new information about the specific questionnaire investigated here but also new information about the generalisability of results pertaining to patient satisfaction questionnaires in general.

One limitation of the study is that the investigated medical conditions and countries have not been selected at random from the universe of all medical conditions and countries. Hence, it is difficult to judge to which extent and in which way the results found here can be generalized. A further limitation of the study is that only 21.9% of the persons approached for participation could be included in the final analyses. Such a small exhaustion rate constitutes a high risk that percentages and means determined from these data deviate from those means and percentages which would have been obtained for the total sample. However, relationships between variables can often be expected to be similar for responders and non-responders. Hence, the low exhaustion rate will most probably not constitute a great danger for the validity of the analyses regarding the central research questions considered here.

### 4.2 Relationships between the SERVQUAL items

A major part of the analyses presented here addresses the relationships between SERVQUAL items. All six basic items correlate positively with each other in all investigated partitions of the data set (see Table 5). Considering that in an ideal index measurement instrument all items should be independent from each other [49], the correlational pattern found here is not desirable. One reason for the high positive inter-correlations might be that all health care providers will, if possible, try to affect all satisfaction relevant characteristics likewise. Hence, these characteristics usually correlate with each other because they are affected by common third variables. This effect will presumably always be present and, thereby, preclude achieving independence between the items. Perhaps, due to this effect, much less dependence than that found here will hardly be possible.

A second reason for the lacking independence of the items might be that, although the items describe possible causes of patient satisfaction, there can also be a causal effect from patient satisfaction on the responses to the items. There might be a so-called ‘halo effect’. The most frequent expression of this effect is that persons with a general positive feeling towards a given object usually bias their judgments of specific characteristics of this object in a positive direction whereas persons with a general negative feeling towards this object do the opposite. This effect produces positive correlations. In index measurement, halo-effects are not welcome as they reduce the extent to which the responses to the items give information about the objective characteristics. Therefore, the items of patient satisfaction indices should be formulated so clearly that they can be answered without resorting to general impressions. This would reduce halo-effects, although it is unlikely to avoid them completely. For this reason, they should be taken into consideration when data are interpreted.

The correlations between the six basic items contain some evidence that the responses to the items are not only produced by halo-effects, but that they actually reflect the characteristics to be judged. Those items which address closely associated characteristics correlate more with each other than items which do not have such closely associated characteristics. For example, empathy and communication are two characteristics which usually are very closely associated. People who feel empathy towards their interaction partner will try to communicate as correctly as possible and, on the other hand, this type of communication presupposes a certain degree of empathy. This relationship corresponds very well to the correlational patterns. The correlation between empathy and communication is highest not only within the total sample but also within nine of the 12 combinations of medical condition and country (see Table 4). On the other hand, the way in which persons interact with each other is only determined by the physical environment to a moderate degree whereas the different aspects of the interaction mostly depend on each other. This also corresponds very well to the correlational patterns. The correlations of assurance, empathy and communication with tangibles are not only the lowest in the total sample; they all also belong to the five lowest correlations in 10 of the 12 combinations of medical condition and country.

The NEDCs reveal that the different item-inter-correlation-matrices are by and large very similar. This is in line with the different effects just discussed. On the other hand, the variance-covariance-matrices which belong to the item-inter-correlation-matrices all differ from each other with a very high level of statistical significance. This reflects that the items relate in a different way to each other in the different contexts. The NEDCs suggest that the differences between the health care given in different countries for the same medical condition are larger than the differences between the health care given for different medical conditions within the same countries. This holds true even when these medical conditions have such different characteristics as diabetes (a chronic medical condition requiring long-time care intervention), and stroke (a sudden traumatic event requiring a direct and fast reaction). This finding suggests that the constraints imposed by the country specific health care systems and health care cultures are stronger than the constraints imposed by the medical conditions to be cared for.

Altogether, the pattern of similarities found here suggests that item-inter-correlation-matrices for different medical conditions and/or in different countries with a Western health system culture will slightly differ from the matrices found here, but that there will be large similarities. These similarities will presumably be larger between different medical conditions in the same country than between the cares given in different countries for the same medical condition.

### 4.3 Relationships of the SERVQUAL-items with general satisfaction

A further key component of the analyses presented here addresses the relationships of the SERVQUAL-items with ‘general satisfaction’. When ‘general satisfaction’ is regressed to all six basic items in a multivariate regression analysis five of these six items have a statistically significant positive regression coefficient whereas one item, i.e. assurance, has a statistically significant negative regression coefficient. The latter holds true although the bivariate correlation between assurance and ‘general satisfaction’ is positive. Presumably, this pattern of results is mainly an effect of the collinearity of the predictors. This collinearity causes so-called suppressor effects.

To investigate how the collinearity influences the pattern of regression coefficients in the multivariate regression analysis additional computations were performed. To be specific, instead of the assurance item, the items most closely correlated with it were removed in a stepwise fashion. In the order of their correlation with the item ‘assurance’ these were: ‘empathy’, ‘communication’, and ‘responsiveness’. When the item ‘empathy’ is removed the regression coefficient for the item ‘assurance’ in the complete sample remains negative and the deviation from zero remains statistically significant, but the regression coefficient is much closer to zero than when all six items are included. When additionally the item ‘communication’ is removed, the regression coefficient for the item ‘assurance’ becomes slightly positive without deviating from zero in a statistically significant manner. When additionally the item ‘responsiveness’ is removed, the regression coefficient for the item ‘assurance’ is positive and the deviation from zero is statistically significant.

The results just reported suggest that the item ‘assurance’ has, at least, two components. One of these components is, by and large, the same as the core meaning of the items ‘empathy’, ‘communication’ and ‘responsiveness’; the other component reflects whether the respondents overrate the different characteristics addressed by the different items in comparison with their judgments of ‘general satisfaction’. The items ‘empathy’, ‘communication’ and ‘responsiveness’ seem to cover the first meaning component better than the item ‘assurance’ and therefore obtain positive regression coefficients in the regression analysis, whereas the item ‘assurance’ obtains a negative coefficient because mainly its second meaning component becomes effective. Altogether, these results suggest that the item ‘assurance’ should not be applied together with the other 5 item in a common index measurement instrument.

When ‘general satisfaction’ is regressed to those five items which remain when the item ‘assurance’ has been removed, all regression coefficients obtained in the total sample are positive and differ from zero in a statistically significant manner (see Table 6). This result suggests that no further items should be removed. The regression analyses performed with the five remaining items for the individual combinations of medical condition and country show that there are slight differences between the regression coefficients for the two different medical conditions and quite remarkable differences between the regression coefficients for the 6 different countries (see Table 6). This suggests that the individual characteristics of the health care or, respectively, the health care provider are valued differently by people with different medical conditions and, especially, from different countries. For example, tangibles seem to have a huge impact on the ‘general satisfaction’ of the Greek patients whereas this item only produces a suppressor effect for the Spanish patients. On the other hand, reliability only produces a suppressor effect in Greece, while it is the second strongest predictor of ‘general satisfaction’ of the Spanish patients.

In the total sample, the correlation between the sum score for the included five items and ‘general satisfaction’ is 0.618. The corresponding statistics for the individual combinations of country and medical condition range from 0.323 for the stroke survey in Spain to 0.790 for the stroke survey in Greece. To evaluate these results a comparison with results from those few studies is helpful for which the correlation between a sum score and ‘general satisfaction’ was reported [4,17,26]. Albashayreh et al. [2] found a correlation of 0.72 with perception of nursing care quality and of 0.82 with the overall quality of care in the hospital using a sum score based on 17 items, Cimas et al. [4] found a correlation of 0.70 with a sum score based on 10 items, Milutinovic et al. [17] found a correlation of 0.75 with a sum score based on 19 items, and Tso et al. [26] found a correlation of 0.85 with a sum score based on nine items.

All correlations just reported are higher than the correlation found for the total sample in the study presented here. However, in all these cases the sum score is based on more than five items. Accordingly, in all these cases more relevant characteristics could have been addressed by the sum score. Hence, taking the results from these studies as a bench mark for the results obtained with the five-item sum score presented here may be regarded as slightly unfair. In any case, the correlation found for this five-item sum score suggests that this score already covers essential determinants of satisfaction, whereas the comparison with the results from the literature suggests that there might still be further determinants which are not addressed by this score.

## 5 Conclusion

All in all the empirical evidence presented here suggests that the item set which results when the item ‘assurance’ is removed constitutes a quite acceptable universal short patient satisfaction questionnaire. With its five items, it is definitively very short and, in spite of its shortness, it possesses quite an acceptable validity. The latter not only holds for the total sample but also, more or less, for the different country specific samples (with perhaps not such convincing results for the Spain case studies). However, the results for the other five investigated countries justify considering the index based upon the selected five items as universal. However, the fact that the regression coefficients differ between the medical conditions and differ even stronger between the countries means that the sum score should, if possible, not be applied without additional analyses. As soon as the investigated sample is large enough, regression analyses with an item addressing general satisfaction should also be performed. Moreover, the means and standard deviations of the individual items should also be considered. All this information will give more detailed suggestions as to which components of the care should be changed in order to improve satisfaction.

There might, of course, be a better five-item set than that identified here. This would be an item-set for which the corresponding sum score correlates more with general satisfaction for all medical conditions and in all the countries and perhaps an item set for which the regression coefficients differ less between medical conditions and countries than in the study presented here. However, finding such an item set needs much further research. Until there is no five-item selection with a more valid sum score, the five-item selection found here could and should be used when only a very short instrument can be applied. This five-item selection should then be referred to as the SERVQUAL-MOD-5 with ‘MOD’ meaning ‘modified’ and five referring to the number of items.

## Supporting information

S1 File Raw data. Raw data.
(SAV)
